# Adipofascial flap resurfacing of proximal interphalangeal joint after contracture release: Mid-term outcomes

**DOI:** 10.1016/j.jpra.2025.03.012

**Published:** 2025-03-21

**Authors:** Yong Chiang Kang, Che-Hsiung Lee, Yu-Te Lin

**Affiliations:** a Department of Hand and Reconstructive Microsurgery, Singapore General Hospital, Singapore; b Department of Plastic and Reconstructive Surgery, Chang Gung Memorial Hospital, Linkou, Taiwan

**Keywords:** Extensor lag, Flexion contracture, Finger contracture, Hand stiffness, Proximal interphalangeal joint, Adipofascial flap

## Abstract

**Background:**

Posttraumatic proximal interphalangeal joint (PIPJ) flexion contracture is a common but difficult problem. Comprehensive literature is sparse, with inconsistent surgical techniques and outcomes. In this study, we describe in detail the volar approach of stepwise release and evaluate the outcomes of using the proximal interphalangeal joint adipofascial flap (PIPJAF) to cover the volar capsule of PIPJ.

**Methods:**

In this retrospective cohort study spanning over 12 years, we compared 19 patients with PIPJAF and 16 patients without PIPJAF, with a minimum follow-up of 6 months postoperatively.

**Results:**

In the PIPJAF group, there was significant improvement in active flexion arc (70.8°, SD 18.6°) at 6 months, and at 6 months and later significantly better extension lag angle (20.8°, SD 19.2°), improvement in extensor lag angle (29.2°, SD 15.3°), and improvement ratio (0.62, SD 0.33) were observed.

**Conclusions:**

There is a modest mid-term benefit in using the PIPJAF. We propose integrating the volar approach with PIPJAF in suitable patients with adequate adipofascial tissue on the lateral aspect of the finger.

**Level of evidence:**

III – retrospective cohort study.

## Introduction

Proximal interphalangeal joint (PIPJ) flexion contracture is an undesirable outcome of the traumatized hand. The contraction (shortening) of the palmar pericapsular tissues manifests clinically as limited active extension, that is, extensor lag. Additionally, the tethering scar around the flexor tendons and palmar skin contractures limit the gliding of extensor tendons to effect joint extension. This affects the motion arc of the injured digit,^1^ and is often a significant component of global hand stiffness.

Surgical gains are modest and often difficult to sustain beyond six months.^2^, ^3^, ^4^, ^5^ Therefore, most surgeons will first attempt a period of rehabilitation with stretching and splinting. Surgery is indicated when the desired function is not achieved by non-surgical measures, or when the presenting flexion contracture is severe. Several authors have published their preferences for surgical approach and structures for surgical release.^6^^,^^7^ Such studies are difficult to conduct for several reasons including variable structures are involved in the contracture, outcomes are dependent on multiple factors, and patients are often lost to follow-up.

Although the mid-lateral approach is preferred to prevent further scar on the volar structures,^8^ we often find that provides inadequate exposure. Several of our cases had open traumatic injuries with contracted skin and scarred flexor sheaths. These cases necessitate exposure to release or remove the contracted structures, and replacement with healthy tissue to provide a gliding surface. We propose our volar approach, sequence for stepwise release, and investigate the effect of using PIPJ adipofascial flap resurfacing (PIPJAF). We had reported earlier on the PIPJAF technique to reduce postoperative pain and improve early active motion.^9^ We have since expanded on this study to include more patients with longer postoperative follow-up.

## Methods

### Data collection

This is a retrospective cohort study carried out in accordance with the STROBE guidelines. After the institutional review board approval, we retrieved consecutive cases from January 2010 to January 2022 from institutional electronic records.

The inclusion criteria were patients with posttraumatic PIPJ flexion contracture requiring volar plate release in a single digit (index to little finger). A residual flexion contracture of ≥30° after trial of therapy, or severe contracture (≥60°) at presentation were offered surgery. We excluded complex cases requiring joint or tendon reconstruction, PIPJ osteoarthritis, cases requiring only tenolysis, and cases with <6 months’ follow-up.

We collected data on demographics and circumstances of injury to ensure the comparability of both groups.

### Surgical procedures

Surgeries were performed by a highly-experienced hand surgeon who receives referrals of PIPJ contracture from five general hospitals. A residual flexion contracture of ≥30° after trial of therapy, or severe contracture (≥60°) at presentation were offered surgery.

**Figure 1:**
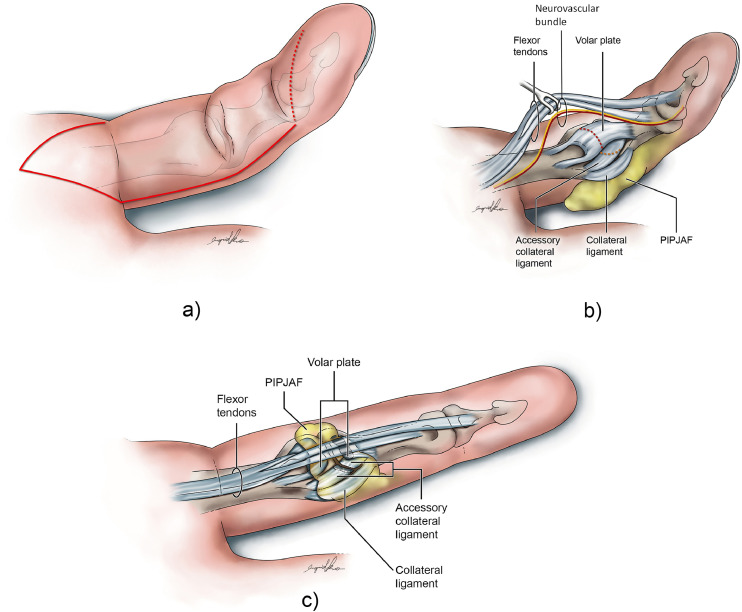
(A) Volar flap is designed to advance the skin and expose the flexor sheath, followed by, as needed, flexor tenolysis and excision of the scarred sheath. (B) Volar plate and accessory collateral ligament are released, and if needed, volar aspect of collateral ligaments (C) PIPJAF is raised from the incised side of the finger and transposed to capsular defect volar to PIPJ, then anchored with absorbable sutures.

We used a volar approach incorporating a volar skin flap. The need for a skin flap depends on the measured shortening as compared to the normal contralateral digit. When skin deficit is 5 to 10 mm, a volar neurovascular advancement flap,^10^ (Figure 1) hinged contralateral to the PIPJAF and modified with a proximal V-Y, is designed. When the skin deficit >10 mm, a free flap such as venous flap is used.

Upon exposing the flexor sheath, the need for flexor tenolysis is determined by observing the gliding of the flexor tendons within the sheath. In some cases, the flexor sheath becomes a cicatrix of thick scar tissue and is excised. Primary reconstruction of pulleys is not attempted. In cases where the flexor sheath is healthy, A3 pulley is excised, preserving A2 and A4 whenever possible (when needed, partial venting is carried out) to gain access to the volar aspect of the joint.

Stepwise release (Figure 1B) is performed until full passive extension is possible without joint buckling—sequentially incising the volar plate transversely, and then releasing the accessory collateral ligament, and collateral ligament if necessary. This is the most sensible, removing one limiting factor (skin, tendon, or periarticular) at a time, and then assessing the degree of motion before moving on to release the next limiting structure.^11^

Once full unresisted passive extension is achieved, the raw surface of the volar PIPJ is considered for PIPJAF whenever possible. This is an intraoperative assessment and relies on the availability of adequate adipofascial tissue on the lateral aspect of the finger.

1) Adipofascial flap is designed with a distally based pedicle adjacent to the PIPJ on its dorso-lateral aspect. Width 5 mm to 10 mm and length 20 mm to 25 mm. The typical length-to-base ratio of the flap is ≤3.
2) Neurovascular bundle is retracted medially and volar-ward.
3) Subdermal plane of dissection from the wound edge, from proximal to distal, using bipolar cautery. The pedicle vessel is not identified routinely but a wide base ensured retrograde perfusion.
4) The pivot point was set proximal to the PIPJ where prominent communicating branches can be preserved.
5) The PIPJ adipofascial flap is then transposed to cover the raw surface on the proximal phalanx head after extension of PIPJ, deep to the flexor tendons.
6) Interrupted sutures with 5–0 nylon were placed starting from the contralateral collateral ligament to the tip of the flap, and anchored with suture to cut ends of the volar plate.

Postoperatively, all patients undergo a standardized rehabilitation regime. The affected PIPJ is splinted in extension with a volar gutter thermoplastic splint, and commenced on interval active and passive range of motion exercises, scar massage, and edema control. After one month, therapeutic ultrasound is used for scar management.

All patients in this study underwent the same perioperative process. Upon clinical consultation or therapy, where possible, the active range of motion was measured using a digital goniometer and recorded electronically. Any complications or recurrence (defined as recurrence of 30° extensor lag) was recorded.

### Statistical analysis

We then analyzed for the differences in postoperative outcome in movement—measuring pre and postoperative extensor lag of the PIPJ, improvement in extension lag angle, and improvement ratio. For the cases with available flexion data, we analyzed the improvement in flexion arc at different postoperative time intervals (6 weeks, 3 months, and 6 months).

As the case numbers were small, we used the Mann–Whitney U Test and Crosstab to analyze the differences between these two groups.

## Results

**Figure 2:**
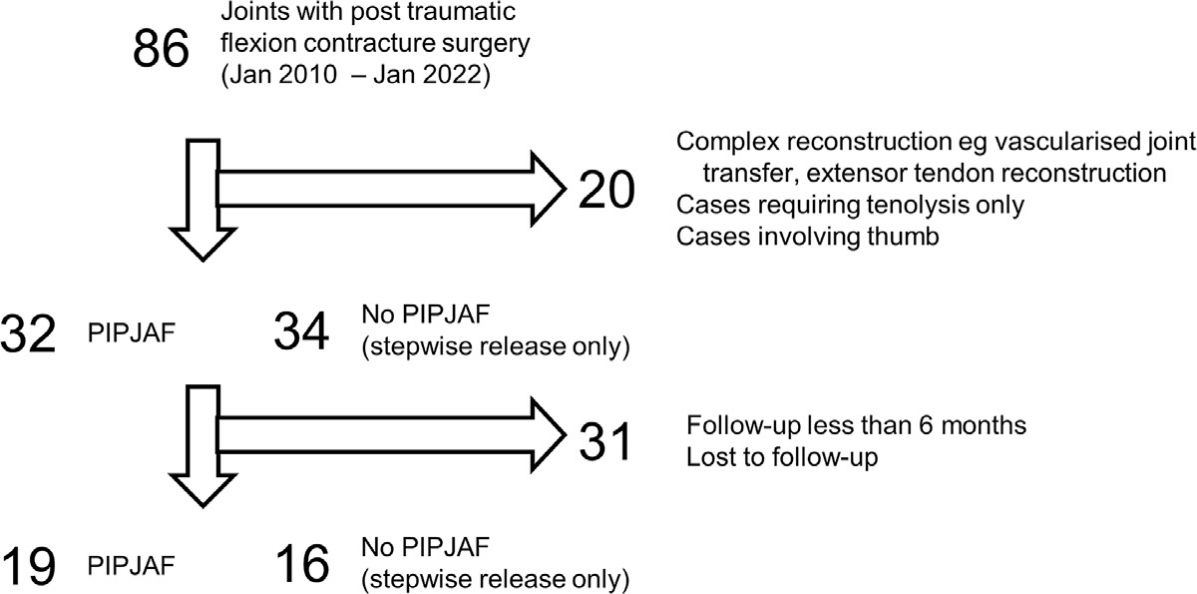
Flowchart demonstrating study inclusion and exclusion.

In the 12-year period, 86 patients were reviewed (Figure 2). Among them, 35 included patients were followed up for more than 6 months (range 183 to 1359 days), and reflected a more sustained outcome of the intervention. Nineteen cases received stepwise release with PIPJAF and 16 cases without PIPJAF.

Most cases in the No PIPJAF group predated the introduction of the adipofascial flap in the year 2016. Thereafter, all cases received the flap unless there was inadequate soft tissue on the lateral aspect of the finger—either an inherently slender finger or scar tissue due to the initial injury.

### Baseline registration

The two groups were comparable in patient demographics, mechanism of injury, and timing of surgery (Table 1). The mean operative age was approximately 40 years (range 10 to 69 years). All patients are cognitively intact and could participate in rehabilitation. The time of injury to contracture release was 31 months (range 6 to 360 months; median 9 months) for the No PIPJAF group, and 20 months (range 6 to 120 months; median 11.5 months) for the PIPJAF group. Patients who required repair of the lacerated structures underwent the surgery at the initial presentation—most patients had only one such surgery before presenting for contracture surgery.

**Table 1:** Baseline registration.

Variable		No PIPJAF (Step-wise release) *n* = 16		PIPJAF *n* = 19		*p*-value
Patient demographics						
Age, mean (years){SD}		41.25	{13.41}	40.00	{18.23}	0.654
Gender, n [%]	Male	10	[63 %]	7	[37 %]	0.130
	Female	6	[37 %]	12	[63 %]	
Side	Left	7		11		0.696
	Right	9		8		
Digit involved, n						
	Index	4		3		
	Middle	2		5		
	Ring	4		5		
	Little	6		6		
Compliance with post-surgical rehabilitation^a^ n, [%]						
	Good	8 [89 %]		7 [64 %]		0.194
	Poor	1 [11 %]		4 [36 %]		
Injury characteristics						
Number of prior surgeries, mean {SD}		1.19	{0.75}	0.95	{0.85}	
Time from injury to current surgery, median {range}		9 months	{6–360}	11.5 months	{6–120}	0.218
Mechanism of trauma						
Sharp injury		3		7		
Blunt contusion		2		3		
Crushing		3		5		
Amputation		4		2		
Unknown		4		3		
Associated injuries						
Dislocation of PIPJ		0		2	[11 %]	0.496
Fracture of proximal or middle phalanx		9	[56 %]	5	[26 %]	0.072
Tendon laceration		6	[38 %]	6	[32 %]	0.506
Nerve injury		5	[31 %]	3	[16 %]	0.187

a Compliance to rehabilitation is regarded as good when patient attends at least one therapy session per month for the first three months.

All patients underwent a period of preoperative rehabilitation to optimize the finger motion, either in the initial treating hospital or at our institution. The degree of compliance to post surgery therapy was similar, as reflected by the patients attending therapy sessions consistently for at least once monthly in the first 3 months.

### Results

The pre and postoperative degree of extension lag is represented in Table 2. There was no significant difference found in preoperative extension and flexion between the two groups.

**Table 2:** Results.

Variable			No PIPJAF (Step-wise release only)			PIPJAF			*p*-value
			n	Mean	SD	n	Mean	SD	
Preoperative extension lag			16	52.5	23.9	19	50.0	16.4	0.973
Preoperative active flexion			7	85.0	19.4	8	83.1	12.5	0.446
Follow up period (days)			16	425 (median 261)	385	19	313 (median 218)	196	0.123
Postoperative extension lag									
	6 months and later		16	36.6	26.1	19	20.8	19.2	*0.034*
	At 6 month		13	25.8	18.1	14	16.8	13.2	0.196
	At 3 month		13	25.8	19.0	12	14.6	17.5	0.118
	At 6 weeks		12	18.3	14.2	15	19.0	15.1	0.842
Improvement in extension lag			16	15.9	23.3	19	29.2	15.3	*0.039*
Improvement ratio			16	0.26	0.42	19	0.62	0.33	*0.013*
Postoperative active flexion									
	at 6 months		13	77.3	17.2	13	87.3	12.0	*0.078*
	at 3 months		10	77.0	18.6	11	84.6	12.7	*0.225*
Flexion arc (active)									
	at 6 months		13	51.5	26.09	13	70.8	18.6	*0.044*
	at 3 months		10	55.5	21.01	11	68.6	22.6	0.176
Maximum VAS within 7 days after surgery			9	2.22	2.49	17	2.47	1.42	0.620
Complications									
	None		16	12	[75 %]	19	14	[74 %]	0.930
	Skin flap ischemia		16	0		19	1	[5 %]	
	Recurrence^a^		16	4	[25 %]	19	4	[21 %]	
Revision surgery for recurrence			16	1	[6 %]	19	0		0.782

a Recurrence is defined as 30° extensor lag after an initial postsurgical improvement.

The PIPJAF and No PIPJAF groups showed improvement in extensor lag that was sustained for at least 6 months. The PIPJAF group showed greater improvement in extension lag angle (20.2° versus 15.9°) and improved extensor lag ratio (0.62 versus 0.26). The improvement in postoperative extensor lag was statistically significant only at the 6 months follow-up and later. Notably, there was a general trend at 3 and 6 months leading up to this difference, with the earliest six-week measurement favoring the No PIPJAF group.

Only 13 cases on each arm had flexion data at 6 months. This allowed us to demonstrate the improved flexion arc at 6 months (70.8° versus 51.5°) (*p* = 0.044). Hence, the mean active range of motion (ROM) at 6 months was 16.8 to 87.3 for the PIPJAF group compared to 25.8 to 77.3 for the control group. This improvement in PIPJAF group resulted in better ROM for function, and better extended position during the opening phase of the hand.

There were no complications from the harvesting of the PIPJAF. In the PIPJAF group, one patient suffered from free venous skin flap ischemia that healed with dressings. This was the only patient requiring a free flap.

## Discussion

To our knowledge, this is the first detailed description combining the use of adipofascial flap resurfacing of PIPJ and volar skin lengthening, at 6 months. Furthermore, PIPJAF resurfacing of the volar pericapsular defect results in sustained improvement of extensor lag at 6 months and later, and flexion arc at 6 months. This applies to posttraumatic contractures with healthy joints and adequate adipofascial tissue on the lateral aspect of the finger.

With the improvement in surgical techniques and postoperative rehabilitation protocols, we managed to obtain sustainable results from flexion contracture surgery. In contrast to the current 71° improvement, the earliest publications manage a gain in ROM between 8° to 24°, with 50 % losing surgical gains by 6 months.^2^ Previous studies should also be interpreted in the correct clinical context, as studies such as the one by Bruser et al. included patients with Dupuytren contracture which has a different pathology and clinical course from posttraumatic contracture.^12^

We use a volar approach to directly evaluate and surgically correct the structures contributing to flexion contracture. The skin tends to be under tension after contracture release, and having incisions over the foci of adhesions (as with Z-plasties, Y-V plasty, or laterally-based transposition flaps) can further limit tendon gliding. To navigate these issues, we prefer a volar neurovascular advancement flap for most cases with measured skin deficits (up to 10 mm). In larger deficits that can occur with severe contractures, we recommend using free flaps such as venous flaps or great toe pulp flap.

Our study showed statistical significance late in the postoperative course, that is at 6 months or later. For us, this was a very interesting finding. Both groups of patients started their early postoperative course with the same rehabilitation protocol and similar degrees of extensor lag. However, at 6 months and beyond when patients were discharged from therapy, the effect of the PIPJAF became more evident and emerged as a statistical significant outcome. This suggests that PIPJAF may have a lasting effect on the eventual outcome.

We postulate that the beneficial effect of PIPJAF is due to three factors:

1) Interposition of scarred tissue. When the volar plate is released, the gap created is gradually filled with scar tissue. Having an interposition of vascularized adipose tissue will disrupt the line of pull of the scar.
2) Reduced gliding resistance of flexor tendon movement. The contribution to gliding resistance of each structure surrounding the tendon changes dynamically in each phase of flexion and extension.^13^ Adipofascial tissue contours well into defects—providing a smooth bed for tendon gliding.
3) Reduction in pain in the immediate postoperative period to enable therapy. As the data on the visual analogue scale was lacking, and this observation remains anecdotal. The nerve ending density of the volar plate is significantly higher than the rest of the periarticular tissue.^14^ Capping these mechanoreceptors with adipofascial tissue can theoretically limit the afferent pain transmission.

### Limitations

The retrospective design of the study resulted in inconsistent data capture especially with regards to data on postoperative flexion and VAS. Although the patients with PIPJAF generally report less pain on mobilization in the immediate postoperative period, could not demonstrate that in this study. There was no formal randomization process in forming the two groups. The decision for using PIPJAF depended largely on the adequacy of the available adipofascial tissue on the lateral aspect of the finger and this was assessed intraoperatively. Furthermore, several of the PIPJAF cases occurred in years after 2016, when the senior author noted a potential benefit; hence, the two groups were largely separated by time. Although the surgical approach is similar between the patients with PIPJAF and without PIPJAF, some patients are not suitable for PIPJAF—because some patients have excessively slender fingers, and others have traumatized and atrophic tissue in the area from which the PIPJAF is harvested.

Patients with flexion contracture are often blue-collar workers and tend to be lost to follow-up during the study period—it took 12 years of clinical cases to draw meaningful conclusions. We arbitrarily included patients with 6 months or longer follow-ups as this reflects a more sustained outcome of the intervention, and also the reality of clinical practice in this patient group. Even with 35 patients, this study remains underpowered. Power analysis showed 32 on each arm will be ideal to demonstrate statistical significance. These cases are rare and tend to be lost to follow-up. Thus, it may be impossible to investigate the long-term outcomes of using PIPJAF.

One may question the viability of the harvested adipofascial tissue given the length-to-base ratio of three or more, especially since the base of the flap does not pivot on a described axial vessel. Digital artery perforators course dorsally from the proper digital arteries at the mid-shaft or phalangeal neck of the middle phalanx. From clinical experience, these vessels support a good longitudinal length of skin^15^ or adipofascial^16^ flap tissue. Lateral designs of cross-finger flap^17^ have been described as well. The circulation is further augmented with radial-ulnar communicating “choke vessels”^18^ that should mature with time.

For future research, the effect on gliding resistance of the tendon can be further evaluated using high frequency ultrasound in a water-bath. There remain challenges in quantifying the measurable outcomes in fingers of different lengths, and different degrees of pulley preservation after trauma.
